# An Overview of the Potential Role of Nutrition in Mental Disorders in the Light of Advances in Nutripsychiatry

**DOI:** 10.1007/s13668-024-00520-4

**Published:** 2024-02-08

**Authors:** Nursel Dal, Saniye Bilici

**Affiliations:** 1 Department of Nutrition and Dietetics, Bandirma Onyedi Eylul University, Balikesir, Turkey; 2 Department of Nutrition and Dietetics, Gazi University, Ankara, Turkey

**Keywords:** Nutritional psychiatry, Nutripsychiatry, Mental disorders, Diet

## Abstract

**Purpose of Review:**

As research on the potential impact of nutrition on mental disorders, a significant component of global disability continues to grow the concepts of “nutritional psychiatry, psycho-dietetics/nutripsychiatry” have taken their place in the literature. This review is a comprehensive examination of the literature on the the potential mechanisms between common mental disorders and nutrition and evaluates the effectiveness of dietary interventions.

**Recent Findings:**

Inflammation, oxidative stress, intestinal microbiota, mitochondrial dysfunction, and neural plasticity are shown as potential mechanisms in the relationship between mental disorders and nutrition. As a matter of fact, neurotrophic factors, which make important contributions to repair mechanisms throughout life, and neuronal plasticity, which plays a role in mental disorders, are affected by nutritional factors. In metabolism, the antioxidant defense system works with nutritional cofactors and phytochemicals. A balanced, planned diet that provides these components is more likely to provide nutrients that increase resilience against the pathogenesis of mental disorders.

**Summary:**

Nutrition can be considered a risk factor for mental disorders. Therefore, developing public health strategies focused on improving diet may help reduce the global burden of mental disorders and other related diseases.

## Introduction

Good mental health is a fundamental aspect of overall well-being. The World Health Organization (WHO) defines mental health as “a state of well-being in which each individual realizes his or her potential, is capable of coping with the stresses of life, can work productively and usefully, and can contribute to his or her society” [1]. Mental health problems are on the rise globally and in Turkey, affecting people of all age [2]. Mental disorders’ impact is steadily increasing worldwide, affecting health and various social, human rights, and economic aspects significantly. Globally, an estimated 322 million people are affected by depression, 50 million by dementia, 45 million by bipolar disorder, and 20 million by schizophrenia, accounting for a significant portion of global disability [3, 4].

Nutrition is considered an important factor in the development of brain function and mental disorders in the context of climate change, urban growth, cultural and technological changes, and industrialization and over-processing of food [5•]. Indeed, recognizing that diets and complementary foods contain essential components and phytochemicals with the potential to impact brain and mental health has given rise to the concepts of ‘nutritional psychiatry/psycho-dietetics/nutripsychiatry’ in the literature [2, 5•, 6, 7]. These concepts are fields of science that seek to understand the mechanisms underlying the effect and potential application of diet for the modulation of specific neurobiological pathways, the use of selected nutraceuticals to correct nutritional deficiencies, and the effects of the quality of various nutrients on mental health [8, 9]. It is widely accepted that a balanced diet is crucial for an individual’s overall health, affecting both physical and mental well-being [10, 11]. For this reason, some modifications are made in the diet and the immune system is regulated with foods that increase resistance against the pathogenesis of mental disorders. Indeed, the antioxidant defense system works with the support of nutritional cofactors and phytochemicals [8]. In addition, neurotrophic factors, which contribute throughout life to neuronal plasticity and repair mechanisms that are important in the development of mental disorders, are affected by nutritional factors [12]. Given the widespread occurrence of mental disorders and the potential advantages of a balanced diet, nutrition is underscored as being crucial not just for psychiatry but also for fields like cardiology, endocrinology, and gastroenterology [7].

This review aims to examine the potential mechanisms between common mental disorders and nutrition in line with current literature information and to evaluate the effectiveness of dietary interventions in light of developments in nutritional psychiatry/nutripsychiatry.

## Mental Disorders

Mental disorder is defined as a “harmful dysfunction” because it reflects an individual’s inability to perform a naturally evolved function, resulting in adverse consequences for that individual [13]. The National Institute of Mental Health (NIMH) has stated that mental illnesses are brain disorders and that, unlike neurological disorders with identifiable lesions, mental disorders should be considered as disorders of brain circuits [14]. More recently, mental disorder has been defined as “a syndrome characterized by clinically significant disturbances in an individual’s cognition, emotional regulation, or behavior that reflects a dysfunction in the psychological, biological, or developmental processes underlying mental functioning” [15].

Although mental disorders are quite diverse, each disorder is described by a set of interrelated symptoms. Moreover, symptoms are not specific to any particular disorder, and the mechanisms underlying these disorders are not fully understood. For this reason, the classification of mental disorders is quite difficult [16, 17]. DSM-V mental disorders are as follows: neurodevelopmental disorders (autism, hyperactivity, etc.), schizophrenia spectrum and other psychotic disorders, bipolar and related disorders, depressive disorders, anxiety disorders, obsessive-compulsive and related disorders, trauma and stress-related disorders, dissociative disorders, somatic symptom, and related disorders. It is divided into headings such as feeding and eating disorders, sleep-wake disorders, and personality disorders [15].

Mental disorders affect individuals across all populations and age groups, making a significant contribution to the overall burden of disease. It has been reported that 80% of those affected by mental disorders reside in low- and middle-income countries [18]. According to the WHO, the most prevalent mental disorders are depressive disorders and anxiety disorders [4], which rank as the leading causes of global disability [3, 19]. Depressive disorders encompass major depressive disorder and dysthymia, while anxiety disorders include generalized anxiety disorder, panic disorder, various phobias, social anxiety disorder, obsessive-compulsive disorder, and post-traumatic stress disorder [15]. The worldwide number of individuals living with depression is estimated to be 322 million, which constitutes 4.4% of the global population. Additionally, it is reported that approximately 264 million individuals live with anxiety disorders, accounting for 3.6% of the global population. Both depression and anxiety disorders are more prevalent in women compared to men. The prevalence of depression varies by age; it can manifest in children and adolescents under 15 years of age but peaks in older adulthood, affecting over 7.5% of women and over 5.5% of men aged 55–74. While the prevalence of anxiety disorders does not exhibit significant variations among different age groups, it is less prevalent in older populations [4].

## Potential Nutrition-Related Mechanisms in Mental Disorders

**Fig. 1:**
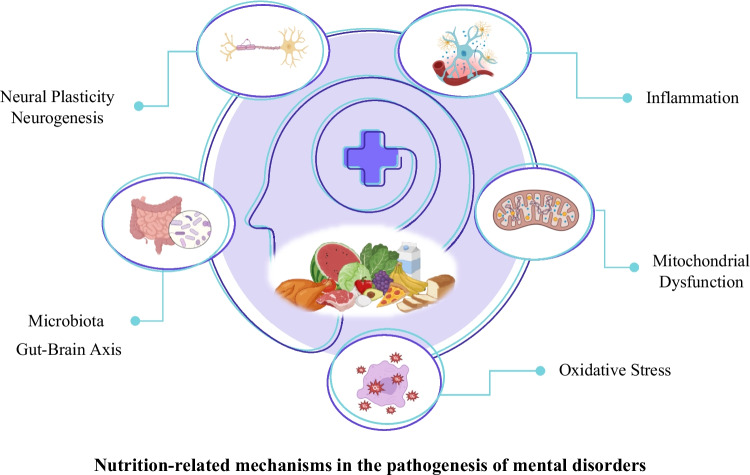
Nutrition-related mechanisms in the pathogenesis of mental disorders

While the mechanisms contributing to the pathogenesis of mental disorders are not yet fully understood, potential nutrition-related mechanisms include inflammation, oxidative stress, intestinal microbiota, mitochondrial dysfunction, and neural plasticity (Fig. 1) [6, 8].

### Inflammation

Chronic low-grade inflammation, characterized by an increase in pro-inflammatory cytokines and acute phase proteins, plays a role in the development of de novo depression, schizophrenia, and bipolar disorder [20–22]. Meta-analyses have shown that mental disorders, including major depressive disorder, bipolar disorder, and schizophrenia, are associated with increased levels of both peripheral inflammatory markers [23] and systemic inflammation [24].

While the factors contributing to inflammation in the development of mental disorders may vary among different disorders, they often encompass lifestyle elements such as psychological stress, smoking, obesity, insomnia, and malnutrition [20]. Diet and food intake can affect the expression of inflammatory biomarkers that have an effect on neuroinflammation [25]. Dietary patterns rich in refined starch, sugar, and saturated and trans fatty acids, but low in polyphenols, fiber, and omega-3 fatty acids, may lead to the activation of the innate immune system with reduced production of anti-inflammatory cytokines and excessive production of pro-inflammatory cytokines [25–27]. Additionally, high-glycemic-load foods and processed meat products have also been associated with the production of inflammatory biomarkers [28, 29].

In a large-scale study, it was found that individuals diagnosed with major depressive disorder (*n* = 14,619), bipolar disorder (*n* = 952), and schizophrenia (*n* = 262) had significantly higher macro and micronutrient intakes compared to healthy controls (*n* = 54,010). Notably, individuals with mental disorders were reported to consume significantly more carbohydrates, sugar, fat, and saturated fat [30]. A meta-analysis studying the relationship between diet, inflammation, and mental health revealed that individuals following a pro-inflammatory diet were 1.4 times more likely to be diagnosed with depression or exhibit depressive symptoms compared to those adhering to an anti-inflammatory diet [31]. Increased risks of mental disorders have been associated with dietary patterns like the Western diet, characterized by high saturated fat and refined carbohydrate intake [32, 33]. In contrast, cohort studies have indicated that dietary patterns such as the Mediterranean diet, rich in fruits, vegetables, olive oil, and legumes, may have a protective effect against mental disorders [34, 35]. Given the findings from these studies and the role of diet in regulating inflammatory processes, dietary modifications appear to play a crucial role in reducing the heightened inflammation observed in individuals with mental disorders [31, 34, 35].

### Oxidative Stress

Normal metabolism produces oxygen-containing reactive chemical molecules, often called reactive oxygen species (ROS), and under normal conditions, these molecules are rendered harmless by the antioxidant defense system [36]. Oxidative stress refers to an imbalance between the cellular production of these reactive oxygen species and antioxidant mechanisms [37]. Inflammatory responses to any infection trigger ROS production in the body, and therefore, it is stated that there is a close relationship between inflammation and ROS formation [38]. Damage caused by oxidative stress and ROS is also related to mental disorders [36]. Oxidative stress parameters and antioxidant capacity, along with low-grade inflammation, have been reported to potentially play a role in many non-communicable diseases, including mental disorders [39, 40].

Various studies show that oxidative and nitrosative stress plays a role in the pathophysiology of major depression [41, 42]. It has been shown that in major depression, increased levels of ROS and peroxide, and altered levels of antioxidant defenses such as glutathione (GSH) [42, 43]. In a postmortem study evaluating the relationship between mental disorders and oxidative stress markers by lipid peroxidation product in postmortem tissue, the oxidative stress marker was significantly increased by approximately 50% in patients with bipolar disorder and schizophrenia and a 33% increase, although not significant, in patients with major depressive disorder. It was concluded [44]. It has been reported that higher levels of oxidative stress markers as well as lower antioxidant levels and antioxidant enzyme activity such as vitamin E, vitamin C, coenzyme Q10, and glutathione are observed in depressed individuals compared to healthy controls [40]. A meta-analysis study including 115 studies examining oxidative stress markers in depressed patients supports the fact that serum total antioxidant capacity, paraoxonase, and antioxidant levels are lower in depressed patients, and serum free-radical and oxidative damage product levels are higher than in control groups. It has been reported that depressed patients have lower antioxidant capacity and serum albumin, HDL cholesterol, and zinc levels during acute attacks [45].

There is inconsistency in the results for total antioxidant capacity and glutathione peroxidase activities in patients with schizophrenia. Some studies have reported that total antioxidant capacity and glutathione peroxidase activities are reduced in schizophrenia patients [46, 47]. In addition, some studies have reported that there is no significant difference in total antioxidant capacity and glutathione peroxidase activities in schizophrenia patients [48, 49] or an increase in total antioxidant capacity and glutathione peroxidase activities [50]. In a recent study, it was reported that only serum glutathione peroxidase activity decreased significantly in schizophrenia patients compared to control groups, and total antioxidant capacity, superoxide dismutase, and malondialdehyde levels did not show significant differences between cases and controls [51].

Diet and some of its components can affect both the intensity of oxidative damage and antioxidant mechanisms [52]. Considering the abundance of antioxidant compounds found in foods such as fruits and vegetables, oxidative stress, which plays a role in the pathogenesis of mental disorders, is one pathway that can be modulated through diet [6].

### Mitochondrial Dysfunction

Mitochondria are intracellular organelles that are very important in cellular energy production [53]. Mitochondrial oxidative phosphorylation is the major ATP-producing pathway, providing more than 95% of the total energy needed in cells [54]. Tissues with high energy needs, such as the brain, contain many mitochondria and are therefore more sensitive to a decrease in aerobic metabolism [55]. At the same time, mitochondria play an extremely important role in many neuronal functions such as synaptic transmission, Ca^+2^ signaling, production of action potentials, and ion homeostasis [56–60]. Therefore, it has been suggested that damage to the mitochondrial electron transport chain is an important factor in the pathogenesis of a number of mental disorders such as bipolar disorder, depression, and schizophrenia [61–63]. Mitochondrial dysfunction is also important in mood disorders due to genetic variation or mutations in nuclear and mitochondrial DNA [53]. In addition, mitochondria contain an extensive antioxidant defense network and multiple electron carriers that can produce ROS [64], and mitochondrial dysfunction can lead to an imbalance in this production, resulting in an increase in ROS production [65]. Therefore, mitochondrial dysfunction has been reported as a source of oxidative stress in both schizophrenia and bipolar disorder [66]. At the same time, the most comprehensively studied disorder related to mitochondria is autism spectrum disorder [35]. It has been reported that both ROS production increases and electron transport chain function is impaired in autism spectrum disorder [67].

The role of nutrients and components in optimizing mitochondrial function is very important. For example, both niacin and riboflavin serve as cofactors in ATP production. Heme iron deficiency causes mitochondrial dysfunction and oxidative stress, and the production of this iron depends on various nutrients such as vitamin B6, copper, and zinc. When all steps of cellular metabolism and energy production are brought together, almost all nutrients and components are involved in modulating mitochondrial function [36]. Additionally, polyphenols (resveratrol, curcumin, epigallocatechin gallate, and quercetin) have been shown to improve mitochondrial functions in different in vitro and in vivo studies [68].

### Microbiota-Gut-Brain Axis

Intestinal microbiota, the diversity and balance of its strains are important indicators for general body health [69]. The data that signals affected by the gut microbiota are transmitted from the gut to the nervous system through the “gut-brain axis,” a bi-directional communication network that includes neural, endocrine, and inflammatory mechanisms, is one of the most important issues focused on in recent years [70]. The gut microbiota has been shown to directly influence neurotransmitter metabolism and have effects on enteric and central nervous system function through the production of molecules such as short-chain fatty acids, secondary bile acids, and tryptophan metabolites [71, 72]. In addition, the gastrointestinal microbiota is associated with various neurobiological pathways related to mental diseases, including modulation of brain-derived neurotrophic factor, serotonin neurotransmission, immune function, and hypothalamic-pituitary-adrenal axis-mediated stress response [71, 73, 74].

It has been stated that there is a possible correlation between intestinal microbiota composition (*Firmicutes*, *Bacteroidetes*, and *Clostridium*) and response to depressive state and chronic stress [75]. Maes et al. [76] suggested that the increased immune response in depression results from bacterial translocation, that is, dysbiosis, which develops with the disruption of mucosal barrier integrity [76]. Studies have shown that there are changes in the intestinal microbiota in clinical depressive disorders compared to healthy control groups, but the findings regarding the diversity of microbial communities in depression are inconsistent [77–79]. It has been determined that microbial richness and diversity are significantly reduced, short-chain fatty acid-producing bacteria are reduced, and bacteria such as *Escherichia-Shigella*, *Fusobacterium*, and *Ruminococcus gnavus* are overproliferated in individuals with generalized anxiety disorder compared to healthy controls [80].

Food type, quality, and source shape the intestinal microbiota profile and affect its function [81]. Studies have shown that higher fiber intake, prebiotics, and probiotics modulate the intestinal microbiota [82, 83]. Additionally, the Mediterranean diet and other healthy dietary patterns rich in plant foods have been associated with increased diversity of the microbiota [84]. In this way, dietary interventions can be considered as a method to be followed in the prevention and treatment of microbiota-related mental diseases.

### Neural Plasticity and Neurogenesis

Neural plasticity is defined as changes in the structural properties and functions of neurons in the brain and the synapses they form, depending on various internal and external stimuli [85]. Neurogenesis is expressed as the formation of new neurons and glial cells and occurs throughout life in certain regions of the brain. It is associated with learning, memory, and mood regulation, especially in the hippocampus [86]. Neurogenesis, which continues into adulthood, is now widely accepted as a fundamental mechanism of neural plasticity [87]. It has been suggested that brain-derived neurotrophic factor (BDNF) and other neurotrophins (e.g., Bcl-2 and vascular endothelial growth factor) mediate hippocampal neurogenesis [88, 89]. Many mental disorders can be defined as maladaptive thought, emotion, and behavior patterns resulting from suboptimal neuroplastic changes that occur at various developmental time points [90]. Neuroplasticity, especially caused by stress, plays a critical role in almost all mental disorders [91].

In recent years, it has been stated that traditional eating habits have been replaced by consuming foods containing saturated fat and sugar and that foods and some nutritional factors contribute to the deterioration of mental health. However, it is not fully understood how nutrients or nutritional factors modulate synaptic function and neuroplasticity [92]. As a matter of fact, clinical research on the effect of nutrition on neural plasticity and neurogenesis is quite limited. Studies in different animal models have reported a positive relationship between omega-3 fatty acids, vitamin E or flavonol intake, and BDNF levels [93, 94]. Diets rich in saturated fatty acids or total fat have been associated with lower BDNF levels, lower neuronal plasticity, and poorer cognitive ability [95, 96]. It has been reported that memory and learning deficits, even with short-term exposure to high-fat/high-sugar diets, are associated with reduced branching, widened synaptic cleft, and synaptic plasticity due to reduced activity in the hippocampus [92]. A diet based on the Mediterranean diet improved plasma BDNF concentrations in individuals with depression [97]. Higher BDNF levels were observed in patients with schizophrenia by increasing the consumption of carotenoid-rich fruits and vegetables [98]. As a result, it can be thought that nutrition is related to neurogenesis and neuroplasticity underlying mental disorders through BDNF levels and possible dietary modifications will be important in treating mental disorders.

## Dietary Interventions for Mental Disorders

In recent years, there has been a growing body of epidemiological studies investigating the link between nutrition and mental health [99, 100]. To safeguard mental well-being, especially in modern societies, dietary modifications such as avoiding processed foods (trans fats and refined carbohydrates and sugars) and returning to the traditional diet that includes the consumption of foods such as vegetables, fruits, seafood, whole grains, lean meat, nuts, and legumes are recommended [8]. The primary rationale behind incorporating such dietary changes and enhancements to bolster resilience against the pathogenesis of mental disorders is that a substantial portion of the total energy and nutrient intake directly supports the human brain. This support includes amino acids, fats, vitamins, minerals, and trace elements, all of which are crucial for both the structure and function of the brain, including intracellular and intercellular communication [2, 7]. The recommended dietary modifications also contribute to the regulation of the immune system, particularly in reducing the risk of depression [8].

Various dietary patterns have been suggested to influence the onset, duration, and severity of mental disorders. Specifically, dietary patterns aimed at reducing inflammatory potential have been linked to a decreased risk of depression [101–103]. For instance, there is a positive association between Western-style dietary patterns and mental disorders like depression and anxiety, whereas Mediterranean diet patterns are reported to have protective effects [99, 104, 105]. Dietary recommendations for reducing inflammation include increasing the consumption of fruits, vegetables, fish, whole grains, legumes, and olive oil, while decreasing the intake of highly refined grains, red meat, fried foods, and sugary treats [106]. Fruits and vegetables, in particular, are rich sources of dietary fiber and antioxidants. Antioxidants play a vital role in shielding cells from oxidative and nitrosative stress, and they contribute to reducing inflammatory potential [107]. Additionally, diets that have been associated with improved mood frequently share common features, such as high intake of unsaturated fatty acids and fiber [108].

While studies investigating the impact of diet on mental disorders generally focus on the correlation between dietary habits and the risk of mental disorders among healthy or overweight/obese individuals [109–113], this review examines dietary interventions specifically for individuals with mental disorders (see Table 1). In a randomized controlled study conducted by Parletta et al. [114], a Mediterranean diet containing fish oil supplements (900 mg/day docosahexaenoic acid and 200 mg/day eicosapentaenoic acid) was applied to individuals with depression aged 18–65 for 6 months and the diet was administered for the first 3 months. Workshops were held every two weeks on how to prepare and cook meals adhering to the Mediterranean diet model. The control group, on the other hand, received no supplements and no training. At the end of the study, the depression subscale scores and mental health scores of the Mediterranean diet group were found to be higher than the control group (1.68 and 1.52 times, respectively), and it was concluded that healthy eating behaviors supported by fish oil could provide improvement in individuals with depression [114]. In a randomized controlled trial aimed at assessing the effectiveness of dietary intervention for treating major depressive episodes, 67 individuals with moderate or severe depression were monitored over a 12-week period. The group that received dietary intervention (*n* = 33) was provided with individualized dietary advice and nutritional counseling support from a clinical dietitian. They also participated in motivational interviewing, goal setting, and mindful eating sessions to encourage compliance with the “ModiMedDiet”—a dietary plan consisting of 11 food groups aligned with recommendations for depression prevention. Each participant in this group received seven support sessions, each lasting approximately 60 min. On the other hand, the control group (*n* = 34) received support from trained personnel on various social topics such as sports, news, or music. At the conclusion of the study, it was found that there was a significantly (*p* < 0.001) greater improvement in depression scores in the group receiving dietary intervention compared to the control group [115].

**Table 1:** Diet intervention studies for mental disorders

**Study**	**Population**	**Method**	**Results**
Parletta et al. [114]	152 individuals with depression between the ages of 18 and 65	• Intervention group (*n *= 75): Mediterranean diet with fish oil supplementation (900 mg/day DHA and 200 mg/day EPA) for 6 months and biweekly workshops on food preparation and cooking for the first 3 months • Control group (*n* = 77): No fish oil supplement or training	The depression subscale scores and mental health scores of the Mediterranean diet group were found to be higher (1.68 and 1.52 times, respectively) compared to the control group. This suggests that healthy eating behaviors, supported by fish oil, can lead to improvements in individuals with depression.
Jacka et al. [115]	67 individuals with moderate or severe depression	• Intervention group (*n* = 33): “ModiMedDiet” (11 food groups) in line with nutritional recommendations for depression prevention for 12 weeks + 7 support sessions, each lasting approximately 60 minutes, focusing on nutrition counseling support, motivational interviewing, goal setting, and mindful eating • Control group (*n *= 34): Support from trained staff on social issues such as sports, news or music for 12 weeks	It was determined that there was a significantly greater improvement in depression scores in the diet intervention group compared to the control group (*p* < 0.001).
Zortea et al. [116]	96 outpatient schizophrenia patients	• Intervention group (*n *= 42): A hypocaloric diet program (low-fat diet containing 20-25 kcal/kg/day) for 6 months • Control group (*n *= 54): A regular diet program without energy restrictions	It has been reported that individuals following a hypocaloric diet had lower serum total radical scavenging antioxidant levels (*p *= 0.022), which may indicate reduced oxidative stres.
Vaghef-Mehrabani et al. [120]	45 women with obesity and major depressive disorder	• 10 g inulin or maltodextrin supplement per day for 8 weeks + an energy-restricted diet • Anthropometric measurements, dietary intakes, depression and levels of zonulin, lipopolysaccharide, inflammatory biomarkers (TNF-α, IL-10, monocyte chemoattractant protein-1, toll-like receptor-4 and C-reactive protein) and BDNF	Body weight (*p *= 0.333) and depression scores (*p *= 0.500) decreased, but the changes were not statistically significant. Furthermore, no significant differences were observed in terms of other psychological outcomes and serum biomarkers (*p *> 0.05).
Freijy et al. [121]	118 adults with moderate psychological distress and low dietary prebiotic intake (< 3 g/day)	• Intervention groups: (1) probiotic supplement and usual diet (probiotic group, *n *= 30) (2) high prebiotic diet (at least 5g/day) and placebo supplement (prebiotic diet group, *n *= 28) (3) probiotic supplement and high prebiotic diet (synbiotic group, *n *= 32) (4) placebo supplement and usual/normal diet (placebo group, *n *= 28)	It was concluded that the prebiotic diet significantly reduced mood disorders compared to the placebo group within 8 weeks (*p* = 0.039). However, it was determined that there was no improvement in symptoms with probiotic or synbiotic treatments (*p *= 0.51 and *p *= 0.92, respectively).

In a cross-sectional study investigating the role of oxidative stress in the pathogenesis of schizophrenia, 96 outpatients diagnosed with schizophrenia were divided into two groups. One group followed a hypocaloric diet program, which included a low-fat diet with a daily intake of 20–25 kcal/kg. The other group adhered to a regular diet program without energy restriction. The study lasted for a minimum of 6 months. At the end of the six-month period, it was observed that individuals on the hypocaloric diet had lower levels of serum total radical scavenging antioxidants, which was suggested to reflect reduced oxidative stress. However, even though the total radical scavenging antioxidant level decreased in the hypocaloric diet group, there was no significant difference in total antioxidant reactivity levels, indicating that the quality of antioxidants remained unchanged [116]. On the other hand, supplements that strengthen the intestinal microbiome and therefore have a positive effect on brain functions have become the subject of research. A meta-analysis of 13 studies comprising 22 treatment and control groups, which evaluated the effectiveness of prebiotics, probiotics, and synbiotics in patients with depression, revealed that patients who received prebiotic, probiotic, or synbiotic treatment experienced a significant improvement in depression compared to the placebo group (standardized mean difference (SMD) =  − 0.34 [− 0.45, − 0.22], *p* < 0.001). However, in three studies with prebiotic intervention, the decrease in depressive symptom scores did not show a significant difference compared to the placebo group (SMD =  − 0.25 [− 0.64, 0.15], *p* = 0.221). Significant beneficial effects on depressive symptoms were observed in subgroups with a treatment duration of less than 8 weeks (≤ 4 weeks: SMD =  − 0.37 [− 0.55, − 0.19], *p* < 0.001; 4 to 8 weeks: SMD =  − 0, 32 [− 0.51, − 0.14], *p* = 0.001) [117].

The presence of prebiotics, particularly, has a therapeutic effect on mental disorders by modulating the balance of gut microbiota and enhancing the number of probiotics in the colon [118]. While further clinical studies are needed, diets rich in dietary prebiotics may be associated with a reduced risk of developing symptoms of psychological disorders [119].

In a study that investigated the effects of supplementing 10 g of inulin or maltodextrin per day for eight weeks in obese and depressed women on an energy-restricted diet, no significant differences were observed between the groups in terms of inflammatory biomarkers (TNF-α, IL-10, monocyte chemoattractant protein-1, toll-like receptor-4, and C-reactive protein) and clinical symptoms, except for depression scores (*p* = 0.005), and other psychological outcomes (*p* > 0.05) [120]. It has been suggested that a high prebiotic diet intervention (at least 5 g/day) can improve mood, anxiety, stress, and sleep in adults with moderate psychological distress and low prebiotic intake (< 3 g/day) [121].

The dietary intervention known as the Mediterranean-DASH Diet Intervention for Neurodegenerative Delay (MIND) is a nutrition model that may have a beneficial impact on mental health due to its antioxidant and anti-inflammatory nature, as well as its reduced consumption of food items such as red meat and sweets [122]. However, when reviewing the literature, the results of studies assessing the risk of depression with the MIND diet are conflicting. In a study conducted over approximately ten years, the Mediterranean diet was associated with a decreased risk of depression (*p* < 0.01), while no significant relationship was found between the MIND diet and the risk of depression. The reduction in depression risk was related to increased consumption of fruits and nuts (*p* = 0.02), moderate nut consumption (*p* = 0.01), and avoidance of fast food/fried foods (*p* = 0.03) [123]. In a study examining the relationship between adherence to the MIND diet and the risk of mental disorders, greater adherence to the MIND diet was reported to be inversely associated with the probability of depression and psychological distress [124]. In an Iranian adult population (*n* = 7165), adherence to the MIND diet was reported to significantly reduce the likelihood of developing depression (OR = 0.62, 95% CI 0.40–0.96; *p* = 0.02) and anxiety (OR = 0.61, 95% CI 0.41–0.91; *p* = 0.01) [125]. A study examining the relationship between the MIND diet, mental health, and metabolic markers in obese individuals associated the highest MIND diet score tertile with lower stress levels and higher insulin sensitivity (*p* < 0.05) [122].

When the literature was examined, it was stated that an effective diet could create potential opportunities to implement pharmacological, therapeutic, and preventive interventions, and it was reported that there were dietary interventions with psycho-protective potential by emphasizing rational nutrition, physical activity, use of psychobiotics, and antioxidant nutrients [5•]. However, it has been reported that a healthy lifestyle, exercise, and dietary changes positively affect insulin regulation in the brain, reduce inflammation and increase BDNF levels [126]. A recent systematic review demonstrated results supporting the positive effects of dietary interventions on body weight management and health outcomes in disorders such as severe mental illness, depression, and anxiety [127••].

## Conclusion

Although most of the mechanisms proposed to explain the potential relationship between nutrition and mental disorders are interrelated, the effect of nutrition on the pathogenesis of these disorders is too important to be underestimated. A healthy, adequate, and balanced planned diet is more likely to provide nutrients that increase resilience against the pathogenesis of mental disorders. As a matter of fact, an adequate and balanced diet is an important component of the treatment applied to support the physical and mental health of individuals living with mental disorders. Especially the Mediterranean diet, with its components, can help prevent and treat mental disorders. Dietitians can contribute to the field of nutripsychiatry by being involved in the implementation of dietary interventions, especially for highly prevalent mental disorders, and by participating in future research in this field, such as diet evaluation and intervention development. Furthermore, considering nutrition as a risk factor for mental disorders and devising public health strategies geared toward improving dietary habits may serve as a crucial step in reducing the global burden of not only mental disorders but also related diseases.
